# The mechanism for exclusion of *Pinus massoniana* during the succession in subtropical forest ecosystems: light competition or stoichiometric homoeostasis?

**DOI:** 10.1038/srep10994

**Published:** 2015-06-05

**Authors:** Junhua Yan, Kun Li, Xingju Peng, Zhongliang Huang, Shizhong Liu, Qianmei Zhang

**Affiliations:** 1 Key Laboratory of Vegetation Restoration and Management of Degraded Ecosystems, South China Botanical Garden, Chinese Academy of Sciences, Guangzhou 510650, China; 2 Hennan University of Animal Husbandry and Economy, Zhengzhou 450046, China; 3 University of Chinese Academy of Sciences, Beijing 100049, China

## Abstract

Competition for light has traditionally been considered as the main mechanism for exclusion of *Pinus massoniana* during succession in subtropical forest ecosystems. However, both long-term inventories and a seedling cultivation experiment showed that growth of mature individuals and young seedlings of *P. massoniana* was not limited by available light, but was strongly influenced by stoichiometric homoeostasis. This is supported by the results of homoeostatic regulation coefficients for nitrogen (*H*_N_) and phosphorus (*H*_P_) estimated using the measured data from six transitional forests across subtropical China. Among three dominant tree species in subtropical forests, *P. massoniana* and *Castanopsis chinensis* had the lowest values of *H*_P_ and *H*_N_, respectively. Therefore *P. massoniana* cannot survive in the advanced stage due to soil phosphorus limitation and *C. chinensis* cannot successfully grow in the pioneer stage due to soil nitrogen limitation. Our results support that stoichiometric homeostasis is the main reason for gradual exclusion of *P. massoniana* from the transitional forest and the eventual elimination from the advanced forest during the subtropical forest succession. Therefore greater attention should be paid to stoichiometric homeostasis as one of the key mechanisms for species exclusion during forest succession.

Competition and facilitation have significant effects on the structure and dynamics of ecosystems and therefore have been a focus in ecology^1^,^2^,^3^. Light competition has traditionally been considered as the main mechanism for tree species elimination from a forest ecosystem. *Pinus massoniana* is an evergreen conifer, native to many regions in the subtropical China (Fig. 1). That species are commonly used as the pioneering trees in forest recovery for degraded ecosystems in the subtropical China. Therefore, *P. massoniana* often dominates the pioneer stage and grows well in the acid red soils^4^. During the forest succession, *P. massoniana* declines in both plant biomass and abundance at the transitional stage, and disappears eventually at the advanced stage^5^. As the pioneer forests are invaded by native broadleaved species, such as *Schima superb* and *Castanopsis chinensis* that often grow faster than *P. massoniana*, and outcompete and eventually exclude *P. massoniana* from the forests, which is traditionally considered being the result of light competition^6^,^7^,^8^. However this has rarely been tested using field observations.

From pioneer to transitional stage, large individuals of *P. massoniana* are initial dominant in the forest ecosystem, which intercept most incoming sunlight. Less sunlight is then available for the other invaded species which grow under the canopy of *P. massoniana* trees. The mechanism of light competition is unlikely to support exclusion of *P. Massoniana* under this condition. The other possible mechanism may be stoichiometric homeostasis, which characterizes the ability of an individual tree in maintaining a relatively steady nutrient balance in a variable environment^9^,^10^,^11^. Previous studies found that there were significant changes in soil biogeochemistry, particularly soil nitrogen (N) and phosphorus (P) availability from pioneer to advanced stage^12^,^13^. If the invaded tree species are more capable of maintaining stoichiometric homeostasis than *P. massoniana* under the soil conditions at transitional or advance stage, they will outcompete and eventually exclude *P. massoniana* during the forest succession.

However these two mechanisms of species elimination operate fundamentally different at ecosystem scale. Competition for light depends on forest structure and shade tolerance of young seedlings, whereas stoichiometric homeostasis represents the ability of an individual organism to maintain its nutrient balance in highly variable soil environments, such as the changes in available soil N^14^,^15^,^16^ and soil P^13^,^17^,^18^ that have been observed during forest succession.

To determine the primary mechanism for the exclusion of *P. massoniana* during a subtropical forest succession, we compiled long-term forest inventory data from the pioneer, transitional and advanced stages at Dinghushan Biosphere Reserve (DBR). These data were used to show the changes in biomass, individual number and tree height of *P. massoniana* during succession. We also collected data from an experiment that cultivated *P. massoniana* seedlings in a large forest gap within the pioneer, transitional or advanced forests at DBR. These data were used to assess whether regeneration of *P. massoniana* was mainly limited by sunlight or stoichiometric homoeostasis. Finally, we collected foliar N and P concentrations and available soil N and P from six transitional forests across subtropical China (Fig. 1). These data were used to calculate the homoeostatic regulation coefficients (*H*)^11^ of the dominated tree species in subtropical forests. The results from the three approaches will be used to explain why *P. massoniana* is gradually excluded from the transitional forest and eliminated from the advanced forest during forest succession.

## Results

### Changes in biomass fraction, individual number and tree height of *P. massonian**a***

In the pioneer subtropical forest ecosystem, *P. massoniana* was the dominated tree species and occupied the entire forest canopy. Biomass of *P. massoniana* accounted for more than 80% of the ecosystem total (Table 1). The decrease in individual number of *P. massoniana* in the pioneer forest was a result of self-thinning, as there was no significant invasion by other tree species into the pioneer forest. During forest succession, the biomass fraction of *P. massoniana* decreased to 62% or 34% in the transitional forest and to zero in the advanced forest (Table 1). However the mean tree height of *P. massoniana* increased as tree grew bigger, and was always greater than other later successional tree species in the transitional forest (Table 1). Therefore above-ground light competition is unlikely to explain why *P. massoniana* decreased its biomass and became extinct in the advanced forest.

### Is the regeneration of *P. massoniana* light limited?

Although above-ground light competition among mature trees cannot explain the decreases in biomass and individual number of *P. massoniana* during forest succession, it is possible that *P. massoniana* species did not successfully regenerate because of light limitation under the canopy, therefore were gradually excluded from the transitional forest as old *P. massoniana* trees died. To test this hypothesis, we cultivated 100 seedlings of *P. massoniana* in a large forest gap receiving full sunlight in each of the three successional forests at DBR over four years (Fig. 2). In the first year, seedlings suffered 7%, 13% and 13% mortality in the pioneer, transitional and advanced forest gaps, respectively. Seedlings mortality rate increased greatly in the second year, with mortality rate of 15%, 44% and 63% in the pioneer, transitional and advanced forest gaps, respectively. In the transitional and advanced forests, all 100 seedlings died by the fourth year, whereas 67% seedlings of *P. massoniana* survived in the pioneer forest at the end of the experiment. Therefore regeneration of *P. massoniana* in the transitional or advanced forest was not limited by light.

As shown in Table 2, measurements of foliar N and P concentrations show that foliar N concentration was highest and foliar P concentration was lowest in the seedlings of *P. massoniana* in the forest gap of the advanced forest. The differences in foliar P concentration between the advanced or transitional forest and pioneer forest were statistically significant (Table 2). As a result, foliar N:P ratio was lowest in the pioneer forest and highest in the advanced forest, and all differences in the seedlings among three forest gaps are statistically significant (Table 2).

### Comparing *H* of three dominant tree species across an environmental gradient

The measured foliar N and P concentrations of *P. massoniana* mature trees and available soil N and P concentrations at six different sites were shown in Fig. 3. Among the three dominated species in the transitional forests, *P. massoniana* had the lowest foliar N and the highest foliar P concentrations. Foliar N concentration of *P. massoniana* varied from 10.05 to 16.68 mg g^−1^, with an average of 12.71 mg g^−1^. Foliar P concentration of *P. massoniana* varied from 0.60 to 0.87 mg g^−1^, with an average of 0.72 mg g^−1^. Comparing with the data in Table 2, foliar N or P concentration of *P. massoniana* mature trees was significantly lower (*p* < 0.05, one-way ANOVA) than that for *P. massoniana* seedlings. The estimates of foliar N:P ratios of *P. massoniana* mature trees varied from 14.70 to 21.61 g N/g P with an average of 17.77 g N/g P. Available soil N and P concentrations, and soil N:P ratio explained more than 80% of their corresponding variations in the needles of *P. massoniana* mature trees across the six forest sites (Fig. 3).

Because foliar N and P concentrations varied significantly among different tree species and between mature trees and seedlings, or different sites for a given species (Fig. 3), foliar N or P concentrations cannot reliably represent the adaptability of different tree species to soils with different available N and P. To account for this variation, we use an index of *H* that measures relative ability of an organism in maintaining its internal nutrient balance in a variable soil environment^11^. By comparing *H* values of different species, we can estimate their ability in adapting to different soil environment. This had been shown for grass ecosystems^19^.

As shown in Fig. 4, *H*_N_ values of *P. massoniana* and *S. superb* are significantly higher than that of *C. chinensis*, and *H*_P_ values of *S. superb* and *C. chinensis* are significantly higher than that of *P. massoniana*. Therefore *P. massoniana* and *S. superb* are much more capable of maintaining their nutrient balance in their needles or leaves and more competitive than *C. chinensis* when N is limiting, and *P. massoniana* is much less competitive than other two tree species, *S. superb* and *C. chinensis*, when P is limiting.

These results are consistent with the observed variations in foliar N and P concentrations based on the seedlings cultivation experiment (Table 2). As found in a previous study^13^, available soil N was lowest and available soil P was highest in the soils of the pioneer forest, and available soil N was highest in the advanced forest, therefore plant growth was most likely limited by available soil N in the pioneer forest soils, and by available soil P in the transitional and advanced forest soils. The lowest *H*_P_ values of *P. massoniana* showed that *P. massoniana* had weak ability in maintaining its internal P balance, and therefore could not survive in the advanced stage due to soil P limitation, while the lowest *H*_N_ value of *C. chinensis* showed that *C. chinensis* had weak regulation ability in its internal N balance, and could not grow competitively in the pioneer forest due to soil N limitation. We must point out that *S. superb* had highest value of *H*_N_ or *H*_P_ in Fig. 4, therefore *S. superb* was very capable of maintaining its internal N or P balance under N or P limiting conditions. This explains why *S. superb* successfully invaded the pioneer forest and also grew well in the advanced forest.

## Discussion

Forest succession generally connotes directional changes in species composition and in community structure over time^20^. The classic theories of succession developed from the observations of temperate ecosystems suggest that light competition or different shade tolerance are the main drivers of invasion and exclusion of different tree species at different succession stages^21^,^22^. However, long-term forest inventories and a seedling cultivation experiment in this study ruled out light competition or light limitation as the main cause for the exclusion of *P. massoniana* during the subtropical forest succession, including mature trees and seedlings. The result is also supported by the long-term inventories of forest gaps^23^ and the observations from other experiments using modelled subtropical forest ecosystems^24^. Inventories of forest gaps found that there were no seedlings of *P. massoniana* in all forest gaps in the transitional or advanced forest^23^. The other experiments also showed that all *P. massoniana* seedlings died in the second year after planting in 10 modelled subtropical forest ecosystems using the soils from the advanced forest^24^.

In this study, we found that stoichiometric homoeostasis rather than light competition was the main mechanism for the exclusion of *P. massoniana* from the transitional or advanced forest. At the pioneer stage, available soil N was limiting and nitrophile species could not survive in those ecosystems. *P. massoniana* occuplied pioneer forests in the subtropical China because of their capability in maintaining internal N balance. During forest succession, soil organic material accumulates in soils and available soil N increases^12^,^13^. As the invaded nitrophile broadleaved species grow, and accumulate both N and P in their biomass, soil available P becomes limiting^13^,^25^,^26^. Therefore, early occupants significantly modified the soil environment, so that further recruitments of *P. massoniana* were much less favored than the late-successional species that were better adapted to the P-limiting soil. Stoichiometric homeostasis that addressed self-limiting in highly variable environmental factors can explain the gradual exclusion of the pioneer tree species, such as *P. massoniana* during the forest succession.

The degree of stoichiometric homoeostasis can explain why *P. massoniana*, the most dominated species in the pioneer forest in subtropical China was eventually excluded from the advanced forest. However, stoichiometric homoeostasis alone cannot explain why *S. superb* with the highest value of *H*_*N*_ was not the pioneer species in subtropical forests, and why the biomass of *S. superb* was lower than that of *C. chinensis* at the advanced stage, even though *S. superb* had the highest value of *H*_*P*_. Light competition, or some other mechanisms rather than stoichiometric homoeostasis alone may explain the position of *S. superb* during the subtropical forest succession^8^.

The drivers of species dynamics during forest succession are complex in a natural environment. The mechanisms of species invasion or exclusion remain controversial. Many previous studies focused their attention on the species dynamics which resulted from competition for the same resource in an ecosystem^27^,^28^,^29^. As a result, competition for resources between plants is considered as the main mechanism in determining the different course of different tree species during forest succession^30^,^31^. More recent studies in aquatic, grass ecosystems, and terrestrial vascular plants quantified the degree of stoichiometric homeostasis^19^,^32^,^33^,^34^,^35^. Our results here support stoichiometric homeostasis as a main mechanism for the exclusion of tree species from forest ecosystems during the forest succession. Therefore more attention should be paid to stoichiometric homeostasis in studying species loss from an ecosystem and in developing a more comprehensive theory of forest succession.

## Methods

### Description of study area

**Figure 1:**
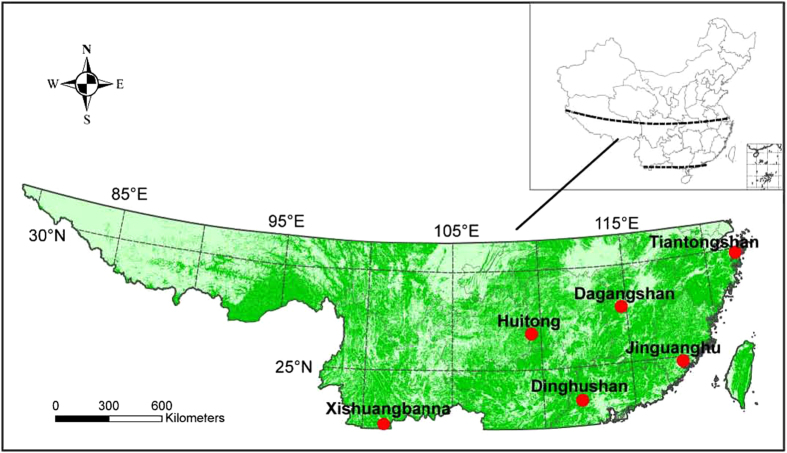
Spatial distribution of the selected six transitional forests across the subtropical China. The data set is provided by Data Center of Resources and Environmental Sciences, Chinese Academy of Sciences (http://www.resdc.cn). Map showing the distribution was made using geographic information system software (ArcGIS version 9.3; ESRI 2012). At Dinghushan site, we have conducted a long-term forest inventory on the dynamics of tree species along the subtropical forest succession gradient since 1978 and an experiment of *Pinus massoniana* seedlings cultivation in the different succession stages since 2010.

**Figure 2:**
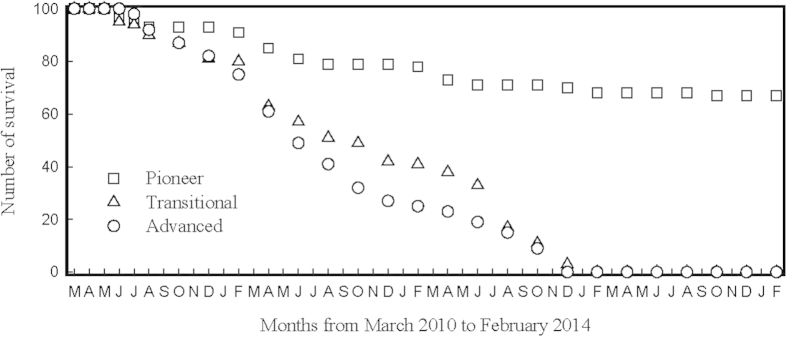
Number of survial *Pinus massoniana* seedlings that cultivated in pioneer, transitional and advanced forest stands along the subtropical forest succession gradient at Dinghushan Biosphere Reserve.

**Figure 3:**
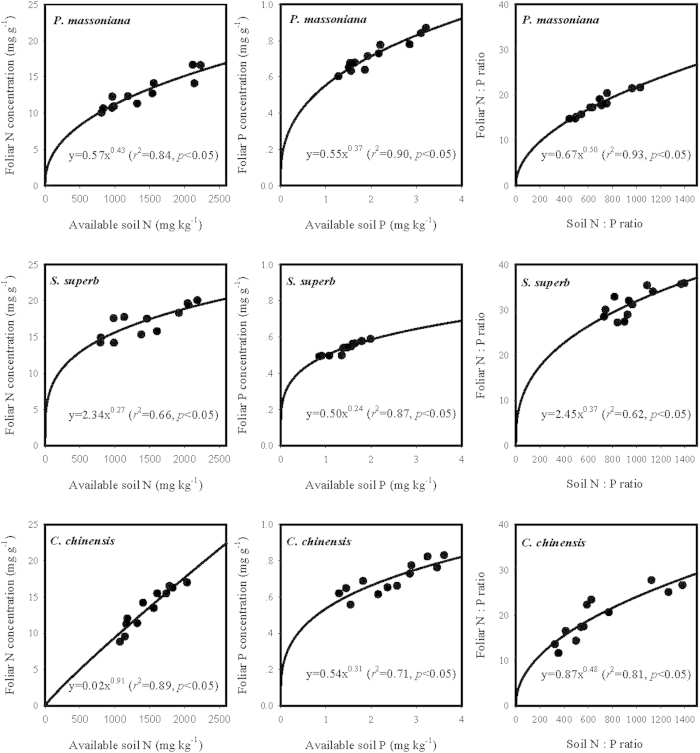
The relationship between foliar N or P concentration (mg g^−1^) or their ratio (Foliar N :P ratio), and the corresponding available soil N or P concentration (mg kg^−1^) or their ratio (Soil N :P ratio) for the three dominated species (*Pinus massoniana, Schima superb* and *Castanopsis chinensis*) in the selected six transitional forests across the subtropical China.

**Figure 4:**
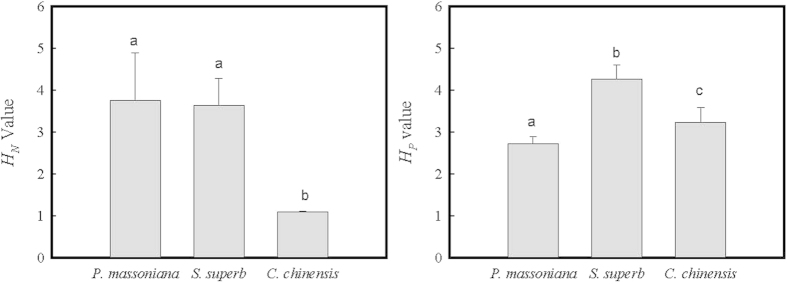
Comparing the homoeostatic regulation coefficients for nitrogen (*H*_N_) and phosphorus (*H*_P_) as calculated using the measured data from six transitional forests across subtropical China. The mean ± standard deviation followed by different lowercase letters has significant differences (*p* < 0.05) among the different tree species by one-way ANOVA.

The subtropical China (Fig. 1) experiences a typical subtropical monsoon humid climate, with a mean annual temperature of 20 °C. The highest and lowest monthly mean temperatures are 28.0 °C in July and 12.0 °C in January, respectively. The average annual rainfall is 1600 mm, of which more than 80% falls from April to September. Three types of natural forest community, i.e., Masson pine forest, coniferous and broadleaved mixed forest and monsoon evergreen broadleaved forest, cover most area of the subtropical China. They were identified by their species composition to represent different successional stages (pioneer, transitional and advanced) along a subtropical forest succession gradient^8^. Permanent plots were established in each of those three forests for studying subtropical forest dynamics and ecological processes (see Fig. 1).

### Long-term forest inventories at Dinghushan Biosphere Reserve

The intact forest stands at DBR (112^°^30^′^39^″^–112^°^33^′^41^″^E, 23^°^09^′^21^″^–23^°^11^′^30^″^N) have not been disturbed in the last 60 years. A long-term project began in 1978 to monitor the dynamics of tree species along the subtropical forest successional gradient. A permanent plot with an area of 1 ha was established in pioneer or advanced forest. The transitional forest has two permanent plots, one is located in buffer area (Plot 1) and the other is located in core area (Plot 2) of the reserve. Plot 2 has not been disturbed longer than plot 1. Height and diameter at breast height of each tree in a plot were recorded and used to estimate the biomass using allometric equations^36^.

### The seedlings cultivation experiment at Dinghushan Biosphere Reserve

Seedlings of tree species *P. massoniana* were collected from an arboretum and grown from seeds until they are two-year-old. The height and basal diameter of all seedlings were very similar. 100 seedlings were transplanted into a large forest gap (>2000 m^2^) in each of the three successional forests (pioneer, transitional and advanced) at DBR over four years. The central region of the gap where seedlings grew received full sunlight for most day time. All planted seedlings received the same tap water during dry seasons, but were not fertilized during the experiment. The number of survived seedlings was recorded monthly during the first 6 months and bimonthly for the remaining months of the experiment. At the end of the second year of the experiment, 10 seedlings were selected and the current-year leaves or needles of those selected seedlings were collected for measuring foliar N and P concentrations in the laboratory.

### Sample collection from the six different transitional forests in the subtropical China

We conducted a field survey of six different transitional forests at Dinghushan, Xishuangbanna (112°32'4''E, 23°10'25''N), Huitong (109°36'12''E, 26°50'18''N), Tiantongshan (121°47'18''E, 29°47'9''N), Jingguanghu (117°54'5''E, 24°54'5''N) and Dagangshan (114°36'50''E, 27°52'13''N), respectively, across the subtropical China in 2008 (Fig. 1). A site was selected in each of six transitional forests that were co-dominated by *P. massoniana*, *S. superb* and *C. chinensis* and were not disturbed for at least 60 years. Samples of current-year needles or leaves were collected from five trees for each of the three species at each site twice a year, one in April and the other in October. Soil samples were collected using a 4.5 cm diameter stainless-steel corer from the top 30 cm mineral soils around the sample trees at each site. Soil samples were taken at four different directions at a distance about 70 cm from the stem of the sample tree, then homogenized into one sample for each sample tree. All foliar or soil samples were placed in sealed plastic bags and taken to the laboratory for analysis.

### Measurements of N and P concentrations

In the laboratory, foliar samples were dried for at least 72 h at 70 °C, then were finely ground. Foliar N concentration (mg g^−1^) was analyzed using the micro-Kjeldahl method. Foliar P concentration (mg g^−1^) was measured photometrically after samples were digested with nitric acid (HNO_3_).

After removing large roots, wood and litter, soil samples were passed through a 2-mm-mesh sieve. Extractable NH_4_^+^ content was determined using the indophenol blue method, followed by colorimetric analysis. NO_3_^−^ content was determined after cadmium reduction to NO_2_-N, followed by sulfanilamide-NAD reaction. Available soil P was extracted with ammonium fluoride (NH4F, 0.03 mol L^−1^) and hydrochloric acid (HCl, 0.025 mol L^−1^), and measured by UV-Vis spectrophotometer (Spectrum lab 24, Shanghai, China). Soil N:P ratio in this study was estimated from the measured total inorganic N (NH_4_^++^NO_3_^−^) and available soil P.

### Homoeostatic regulation coefficient (*H*) calculation

*H* of the three dominated tree species in the transitional forests was calculated using the equation of 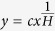
11, where *y* is foliar N or foliar P concentration (mg g^−1^) of sample trees , *x* is available soil N or available soil P concentration (mg kg^−1^) around the sample trees and *c* is a constant. We used the selected six transitional forests across subtropical China (Fig. 1) to provide much larger gradients of N and P in leaves or soils to obtain a more robust estimates of *H* for N or P. In this study, *H* is used to quantify the ability of an organism in maintaining its internal nutrient balance under variable environmental conditions. A species with higher *H* value is more capable of maintaining its nutrient balance and grows better than a species with lower *H* value^11^,^18^.

## Additional Information

**How to cite this article**: Yan, J. *et al*. The mechanism for exclusion of *Pinus massoniana* during the succession in subtropical forest ecosystems: light competition or stoichiometric homoeostasis? *Sci. Rep*. **5**, 10994; doi: 10.1038/srep10994 (2015).

## Figures and Tables

**Table 1:**
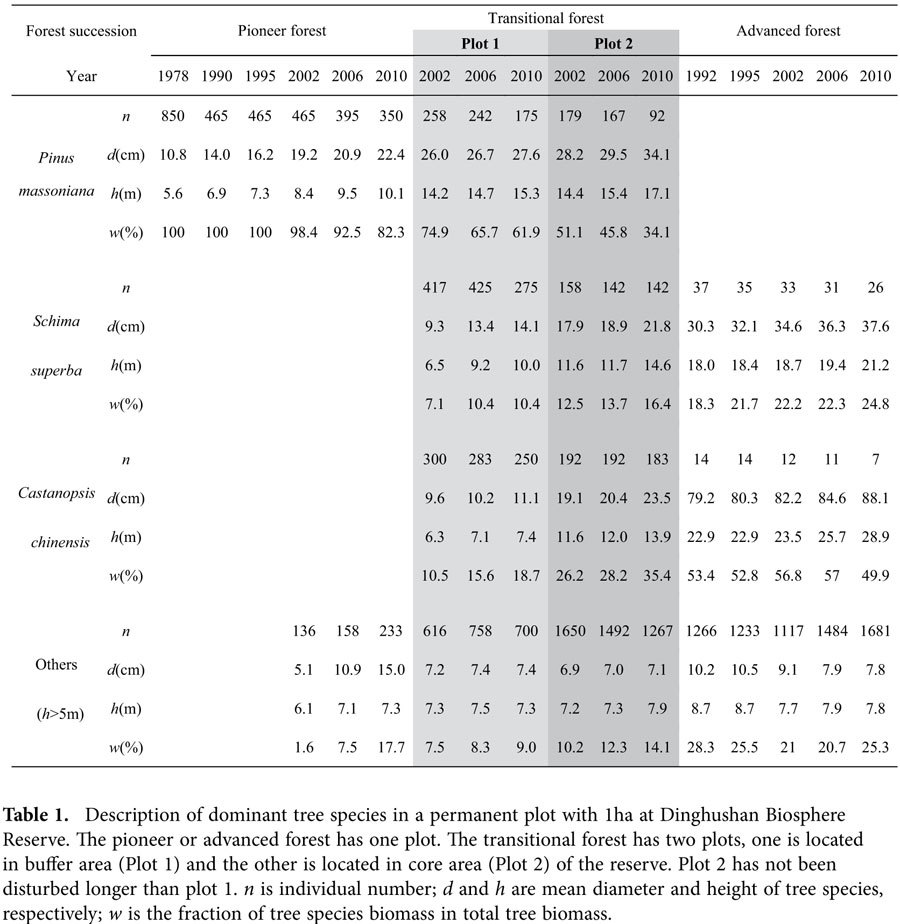
Description of dominant tree species in a permanent plot with 1ha at Dinghushan Biosphere Reserve.

**Table 2:** Foliar N and P stoichiometry of *P. massoniana* seedlings that cultivated in pioneer, transitional and advanced forest stands along the subtropical forest succession gradient at Dinghushan Biophere Reserve.

Forest	N (mg g^−1^)	P (mg g^−1^)	N:P ratio
Pioneer	20.79 ± 1.68^a^	1.43 ± 0.21^a^	14.08 ± 1.86^a^
Transitional	21.54 ± 1.38^a^	0.99 ± 0.08^b^	21.76 ± 1.67^b^
Advanced	22.10 ± 1.30^a^	0.87 ± 0.08^b^	25.40 ± 1.93^c^

The mean ± standard deviation followed by different lowercase letters has significant differences (*p* < 0.05) among the different succession forests by one-way ANOVA.
